# Supplementary material for: Causal Relationship between Aging and Anorexia Nervosa: A White-Matter-Microstructure-Mediated Mendelian Randomization Analysis
Source: Biomedicines. 2024 Aug 16;12(8):1874. doi: 10.3390/biomedicines12081874 (PMC11351342; doi:10.3390/biomedicines12081874)
Supplement: Supplementary file 1 [file biomedicines-12-01874-s001.zip › Supplementary Figure/Figure S9/Leaveoneout plot of MR analysis for ubm-b-1914 to AN.pdf]

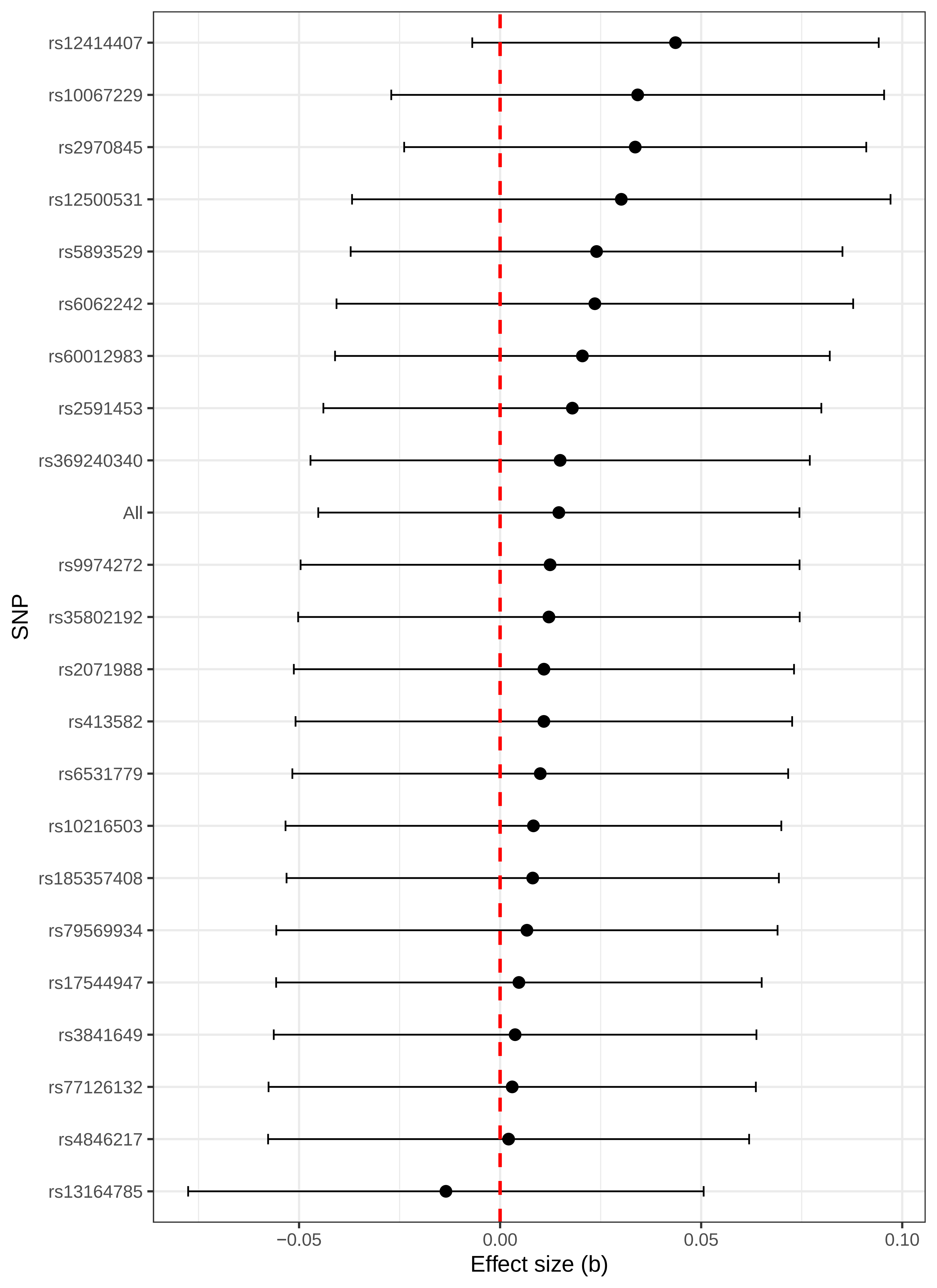
rs12414407
rs10067229
rs2970845
rs12500531
rs5893529
rs6062242
rs60012983
rs2591453
rs369240340
All
rs9974272
rs35802192
rs2071988
rs413582
rs6531779
rs10216503
rs185357408
rs79569934
rs17544947
rs3841649
rs77126132
rs4846217
rs13164785
SNP
−0.05
0.00
0.05
0.10
Effect size (b)
